# Chest physiotherapy protocol based on ultrasonic signs and its preliminary clinical application in patients with severe traumatic brain injury

**DOI:** 10.3389/fneur.2026.1826877

**Published:** 2026-08-20

**Authors:** Qiuyan Shen, Jing Zhang, Yeping Xu, Fengzhi Jiang, Yan Wu, Shibai Sun, Xiaomei Xu

**Affiliations:** 1 Department of Neurosurgery, The 904th Hospital of Joint Logistic Support Force, People's Liberation Army, Wuxi, Jiangsu, China; 2 Department of Nursing, The 904th Hospital of Joint Logistic Support Force, People's Liberation Army, Wuxi, Jiangsu, China; 3 Central Sterile Supply Department, The 904th Hospital of Joint Logistic Support Force, People's Liberation Army, Wuxi, Jiangsu, China

**Keywords:** chest physiotherapy (CPT), Delphi method, evidence-based nursing, severe traumatic brain injury (sTBI), ultrasound

## Abstract

**Objective:**

This study aimed to develop and Preliminary validate an ultrasound-guided chest physiotherapy (CPT) protocol for patients with severe traumatic brain injury (sTBI), offering guidance for clinical pulmonary care management.

**Materials and methods:**

A comprehensive search was conducted across both domestic and international databases from their inception until April 30, 2026, to gather relevant evidence for drafting an ultrasound-guided chest physiotherapy (CPT) protocol for sTBI patients. The draft underwent two rounds of revisions via the Delphi method to finalize the protocol. A convenience sampling approach was adopted to select 20 sTBI patients from a tertiary Grade A hospital in Jiangsu Province, between November 2025 and January 2026, as the experimental group, who received standardized chest physiotherapy (CPT) guided by lung ultrasound assessment. Another group of 20 sTBI patients, treated with the identical standardized chest physiotherapy (CPT) between February and April 2026, served as the control group, and the only difference between the two groups lay in pulmonary evaluation methods: the control group adopted routine pulmonary evaluation including auscultation and CT instead of ultrasound-guided assessment. To compare the pulmonary ultrasound score, clinical pulmonary infection score, oxygenation index, mechanical ventilation duration and NICU length of stay between two patient groups. Additionally, a questionnaire survey of neurointensive care unit (NICU) nurses evaluated the clinical applicability, significance, feasibility, and satisfaction of the protocol.

**Results:**

A total of 34 articles were included, with 18 experts completing two rounds of correspondence. All questionnaires were validly recovered, yielding expert authority coefficients of 0.87 and 0.95. The Kendall harmony coefficients for item importance and operability were 0.129 and 0.137, and 0.305 and 0.244, respectively. The coefficient of variation for each item ranged from 0.048 to 0.221. The final protocol consists of four primary items: personnel qualification, pulmonary ultrasound examination, quality control, and efficacy assessment, along with 11 secondary items and 28 tertiary items. After intervention, statistically significant improvements in lung ultrasound scores emerged from day 7 in the experimental group, while those in oxygenation index reached statistical significance starting on day 9 (*p* < 0.05). Nurses rated the protocol’s clinical applicability, feasibility, and satisfaction at 4.57 ± 0.36 points, awarding a full score of 5 points for its clinical significance, indicating that this protocol has preliminary operability and potential clinical application value.

**Conclusion:**

The ultrasound-guided CPT protocol for sTBI patients is scientifically,potential effectiveness and goal-oriented, providing a valuable reference for pulmonary management in this patient population.

## Introduction

Severe traumatic brain injury (sTBI) refers to significant brain damage resulting from external trauma, often leading to long-term functional impairment and high rates of mortality and disability (1). Due to the brain-lung interaction, sTBI not only disrupts nervous system function but also triggers a cascade of reactions within the respiratory system (2, 3). Pulmonary infection, a common complication, occurs in up to 80% of cases and severely impacts patient prognosis (4). Chest physiotherapy (CPT) refers to a series of physical airway-clearance interventions, including postural drainage, percussion, vibration and respiratory training, aimed at promoting sputum excretion, improving lung ventilation and preventing pulmonary complications (5). Chest physiotherapy (CPT) is widely utilized in clinical practice (6), but its effectiveness is heavily reliant on accurate assessment results. Current clinical evaluations predominantly depend on the expertise and experience of healthcare providers, leading to considerable variability and the potential for inaccurate pulmonary assessments, which can result in deviations from the CPT protocol. Additionally, sTBI patients frequently present with concurrent intracranial hypertension, a condition that complicates movement and transportation, thereby limiting the use of CT imaging (7). Bedside lung ultrasound offers advantages such as being dynamic, real-time, radiation-free, and repeatable, making it a valuable tool for overcoming the limitations of traditional lung assessments. It enables rapid visualization of lung conditions in critically ill patients at the bedside (8). However, existing research mainly focuses on patients with hypostatic pneumonia or atelectasis resulting from prolonged bed rest, while systematic CPT protocols for sTBI patients remain underdeveloped. Pulmonary conditions in sTBI patients often progress rapidly, with insidious onsets (9) and heterogeneous pathology, including edema, atelectasis, and pulmonary consolidation. Given the critical state of these patients, therapeutic procedures are prone to induce fluctuations in intracranial pressure (ICP), making conventional interventions particularly risky (3). Bedside ultrasound allows for real-time, dynamic assessment of pulmonary conditions, facilitating the early identification of lesions before clinical signs of infection appear, and enabling the localization of sputum retention sites for targeted interventions (10). This ensures precise nursing care while maintaining stable ICP (11). In light of this, the study developed an ultrasound-guided CPT protocol for sTBI patients based on evidence-based methodologies, offering a practical reference for clinical practice.

## Materials and methods

### Establish an interdisciplinary research team

The research team comprises 11 members: 1 chief nurse, 4 associate chief nurses, 5 head nurses, and 1 attending physician. Among them, 7 hold master’s degrees and 4 hold bachelor’s degrees. All team members are actively involved in clinical practice, with extensive experience in neurocritical care and related fields. Each member has undergone systematic training in evidence-based methodology and holds specific responsibilities in the project. The chief nurse is responsible for overall coordination and management, while the associate chief nurses assist in revising draft plans, contacting experts for consultations, ensuring quality control, and resolving disagreements during the evidence-based process. The master’s degree holders are responsible for literature retrieval, screening, quality evaluation, evidence extraction, as well as compiling, distributing, and recovering expert consultation questionnaires. They also manage data statistical analysis and contribute to writing the research paper. The project has been registered with the Evidence-Based Nursing Center of Fudan University (ES20258197).

### Developing an initial plan for chest physiotherapy in patients with severe traumatic brain injury under ultrasound guidance

#### Literature review

Search terms in Chinese: craniocerebral injury, craniocerebral injury patients, craniocerebral injury coma, pulmonary rehabilitation, chest physiotherapy, postural drainage, vibration expectoration, ultrasound, ultrasound signs, ultrasonic wave. Search terms in English: craniocerebral injury/patients with craniocerebral injury/coma due to craniocerebral injury/pulmonary rehabilitation/chest physiotherapy/postural drainage/vibration expectoration/ultrasound/ultrasonic. Databases consulted include BMJ Best Practice, UpToDate, Cochrane Library, Guideline International Network (GIN), National Institute for Health and Clinical Excellence (NICE), Registered Nurses’ Association of Ontario (RNAO), Scottish Intercollegiate Guidelines Network (SIGN), Joanna Briggs Institute (JBI) Centre for Evidence-Based Healthcare Database, China Medical Tribune Clinical Guidelines Network, China National Knowledge Infrastructure (CNKI), Wanfang Data, PubMed, Web of Science, Embase. Inclusion criteria: Studies, guidelines, and expert consensus published from database inception to April 30, 2026, concerning patients with a clinical diagnosis of sTBI and involving lung ultrasound/ultrasound signs, critical care ultrasound, CPT, thoracic-pulmonary physical therapy, pulmonary rehabilitation, and pulmonary care. Two researchers, trained in evidence-based medicine, independently assessed the quality of the final selected literature using appropriate evaluation tools. The clinical practice guidelines adopted the internationally recognized AGREE II tool (2009 edition); expert consensus assessments utilized the JBI Evidence-Based Healthcare Center Consensus Study Evaluation Tool; systematic reviews employed the AMSTAR-2 scale (2017 edition); and randomized controlled trials underwent customized bias risk assessments using the JBI Evidence-Based Healthcare Center RCT Evaluation Tool (2016 edition). An evidence-based framework was then developed, summarizing the best available evidence for the following protocol components: personnel qualification access, lung ultrasound examination, quality control, and outcome evaluation. A draft of the ultrasound-guided CPT protocol for sTBI patients was formulated based on this framework.

#### Expert meetings and preliminary plan

1. Expert Meetings: The expert panel, consisting of 11 members—1 chief nurse specialist, 4 associate chief nurse specialists, 5 charge nurses, and 1 attending physician—convened to discuss the protocol. Seven members hold master’s degrees, and four hold bachelor’s degrees. All experts are engaged in clinical practice with substantial experience in neurocritical care. The meeting agenda was determined following a literature review, and each session lasted 1.5 h to 2 h. Researchers strictly avoided leading suggestions, focusing solely on posing objective questions and meticulously recording and organizing the meeting content
2. Developing a Preliminary Plan: Using evidence-based methods, a draft of the ultrasound-guided CPT protocol for sTBI patients was developed following expert discussions. This draft, based on the best summarized evidence, includes four primary components: personnel qualification access, lung ultrasound examination, quality control, and efficacy evaluation. Meanwhile, the intervention operation details were described in strict accordance with the TIDieR guidelines for standardized reporting. Following this, the first round of expert consultation was conducted on the specifics of these four components.

### Expert consultation

#### Selecting experts for inquiries

Inclusion criteria for experts: Active participation in this study; clinical medical experts, clinical nursing experts, ultrasound imaging experts, evidence-based medicine experts, and other professionals with over 10 years of experience; holding associate senior professional titles or intermediate professional titles, with a master’s degree or higher. A total of 18 experts from relevant fields were recruited for written consultation.

#### Conducting expert inquiry

Based on the preliminary plan, a four-part expert inquiry form was developed, consisting of the following components: (1) instructions for completing the form and a letter to the expert; (2) an expert basic information form, including the expert’s name, educational background, age, professional field, and years of experience; (3) a familiarity assessment and judgment basis survey; and (4) a consultation form on the ultrasound-guided CPT protocol for sTBI patients. Experts were asked to rate the importance of various indicators using a 5-point Likert scale, with scores ranging from 1 (Very Unimportant) to 5 (Very Important). Additionally, a section for modification and supplementary opinions was included, allowing experts to propose changes or deletions to the protocol. Expert inquiries were conducted via electronic questionnaires. After obtaining informed consent from the experts, the questionnaires were distributed through channels such as WeChat and email. Each round of questionnaire completion lasted 2 to 3 weeks, with a 4-week interval between rounds. After the first round of responses, the initial entries were revised based on the indicator screening criteria discussed during expert meetings, and the second-round consultation forms were then distributed. This process continued until consensus was achieved among the experts.

### Preliminary application and effect evaluation of the scheme

#### Subject and object of study

A convenience sampling method was employed to select sTBI patients and neurointensive care unit (NICU) nurses from a tertiary Grade A hospital in Jiangsu Province for preliminary validation of the protocol. A total of 20 patients with severe traumatic brain injury (sTBI) enrolled between November 2025 and January 2026 were included as the control group, who received the identical standardized chest physiotherapy (CPT) after routine pulmonary evaluation including auscultation and CT examination. Another 20 sTBI patients enrolled between February and April 2026 were assigned to the experimental group, who received identical standardized chest physiotherapy guided by lung ultrasound assessment. The only difference between the two groups lay in pulmonary evaluation methods: the control group adopted routine pulmonary evaluation including auscultation and CT instead of ultrasound-guided assessment. The only difference between the two groups lay in the methods of pulmonary evaluation, while the subsequent chest physical therapy protocols were consistent in both groups. Inclusion criteria for patients: (1) Age between 18 and 75 years; (2) Clinical diagnosis of sTBI (1); (3) Glasgow Coma Scale (GCS) score of 3–8; (4) Hemodynamic stability with mean arterial pressure > 65 mmHg; (5) No contraindications for chest physiotherapy; (6) Informed consent from the patient or legal representative for voluntary participation in the study. Exclusion criteria: (1) Acute hemorrhagic diseases or pulmonary embolism; (2) Severe chest or abdominal injuries with wounds, drainage tubes, or dressings; (3) Conditions causing restricted body positions; (4) Other factors limiting pulmonary ultrasound examination. After the application of the protocol, nurses from the same NICU were selected to assess the clinical applicability, significance, feasibility, and satisfaction of the protocol. Inclusion criteria for nurses: (1) Currently working in the NICU; (2) At least 3 years of NICU experience; (3) Holding a bachelor’s degree or higher. Exclusion criteria: Nurses undergoing rotations, advanced training, or not on duty during the study period. This study was approved by the hospital’s medical ethics committee (Approval No. 20250601) and is registered with the China Clinical Trial Registry (ChiCTR2500106430).

#### Intervention method

Both patient groups underwent pulmonary ultrasound evaluations after ICU admission. The initial assessment was completed within 6 h post-admission, followed by subsequent evaluations on the mornings of days 3, 5, 7, and 9 before CPT. The experimental group received CPT intervention based on ultrasound assessments, guided by a target-oriented, comprehensive nursing program. In contrast, the control group received traditional CPT measures based on sputum characteristics, auscultation, and results from X-ray or CT examinations.

CPT was performed in strict accordance with standardized operating procedures by ICU nurses who completed unified pre-study training, and intervention details are reported following the TIDieR guidelines. Pulmonary ultrasound was performed by nurses or physicians with Critical Care Ultrasound Study Group (CCUSG) certification using a Philips EPIQ7 ultrasound system with a C5-1 convex probe, following a 12-zone scanning protocol for bilateral lung assessment. CPT was delivered twice daily (09:00–10:00 and 15:00–16:00) for 9 consecutive days, 30–40 min per session, scheduled 30 min before meals or 2 h after meals. Treatment adherence was defined as completion of at least 80% of scheduled sessions and monitored via bedside checklists. Intervention fidelity was assessed by randomly auditing 20% of sessions using a standardized checklist, with ≥90% compliance defined as acceptable.

Pulmonary Ultrasound Examination Method (12): The twelve - segment method was employed. The bilateral chest wall was divided into upper and lower parts at the level of the patient’s nipple. Each side of the chest wall was further segmented into anterior, lateral, and posterior regions based on anatomical landmarks, including the sternum, anterior axillary line, posterior axillary line, and spine. This division results in a total of twelve regions across both lungs. Pulmonary ultrasound scoring system: Scoring was based on ultrasound findings in each region: 0 points: Presence of pleural slippage and A-line; 1 point: Distance between B-lines is ≥ 7 mm (B7 line); 2 points: Distance between B-lines ≤ 3 mm (B3 line); 3 points: Fragmentary or tissue-like signs. The total score was calculated by summing the individual scores from all twelve zones, yielding a score range from 0 to 36. Higher scores indicate more severe pulmonary ventilation defects.

Effect Evaluation: Both groups were followed for 9 days. Pulmonary ultrasound scores, clinical pulmonary infection scores and oxygenation indices were compared on days 1, 3, 5, 7, and 9, while total duration of mechanical ventilation and NICU stay were analyzed as final outcome measures. Additionally, a questionnaire survey was conducted among nurses to evaluate the clinical applicability, significance, feasibility, and satisfaction of the proposed protocol. A 5-point Likert scale was used for the survey, with scores ranging from 1 to 5, where: 1 = “Very unsuitable/very meaningless/very unfeasible/very unsatisfactory”; 5 = “Very suitable/very meaningful/very feasible/satisfactory.”

### Statistical methods

Data entry was performed using Excel 2016 and SPSS 25.0 statistical software, Multiple testing correction was performed using R 4.5.0. followed by descriptive statistics and analysis of experts’ general information and consultation outcomes. All measurement data were first subjected to the Shapiro–Wilk test for normality assessment. Normally distributed measurement data are presented as mean ± standard deviation ( $\bar{x}$ ± s), between-group differences were analyzed using the two-sample t-test. The multiple testing correction was performed using the FDR (Benjamini-Hochberg) method, with *p* < 0.05 considered statistically significant. Categorical data are reported as frequency and percentage (%), between-group differences were assessed using the chi-square test or Fisher’s exact test. Cohen’s d is reported as the effect size alongside the t-value and *p*-value. Specifically, Cohen’s d was calculated as (Control group mean - Experimental group mean)/pooled standard deviation. Lower scores indicate better outcomes for pulmonary ultrasound scoring and clinical pulmonary infection score, while a higher oxygenation index indicates better oxygenation. The magnitude of Cohen’s d was interpreted as follows: |d| < 0.2 = trivial effect, 0.2–0.5 = small effect, 0.5–0.8 = moderate effect, and > 0.8 = large effect. The questionnaire response rate was used to assess expert engagement. Expert credibility was measured using the authority coefficient (Cr), which is derived from the expert judgment basis coefficient (Ca) and expert familiarity coefficient (Cs), with higher values indicating greater authority. Item importance scores and the coefficient of variation (CV) were used to assess the concentration of expert opinions. Kendall’s W coefficient was applied to evaluate the consistency of expert opinions. A *p*-value of < 0.05 was considered statistically significant.

## Results

### Literature search results

A total of 1,033 articles were retrieved from the initial search. After deduplication and preliminary screening of titles and abstracts, 201 articles were selected. Following further screening, 34 articles were ultimately included, comprising 4 guideline (12–15), 9 expert consensus articles (16–24), 2 systematic reviews (25, 26), 14 randomized controlled trials (RCTs) (27–40), and 5 quasi-experimental studies (9, 41–44). Different bias-risk assessment tools were applied for various study designs. Methodological quality varied across included studies, which may weaken the reliability of evidence synthesis. Most RCTs lacked adequate allocation concealment and blinding, possibly resulting in overestimated intervention effects. Some quasi-experimental studies had inconsistent baselines, non-standard controls and incomplete follow-up, causing confounding bias. Several systematic reviews had narrow retrieval ranges and lacked publication bias evaluation, while expert consensus was highly subjective with low evidence level. In conclusion, despite standardized protocol formulation, the robustness of our conclusions was limited by inherent defects in original studies. The ultrasound-guided physical therapy protocol should be applied cautiously in clinical practice. Further high-quality, rigorously designed RCTs are needed to strengthen evidence for its application in sTBI patients.

### Expert consultation results

#### Basic information of inquiry experts

The study involved 18 experts from various regions, including Beijing, Chongqing, Wuhan, Guangzhou, Hangzhou, Wuxi, and Anhui, who were invited to participate in written inquiries. Among the experts, 12 were female (66.7%) and 6 were male (33.3%); 8 held a bachelor’s degree (44.4%) and 10 had a master’s degree or higher (55.6%); 1 was a full professor (5.5%), 13 were associate professors (72.3%), and 4 held mid-level titles (22.2%). The average age was 41.05 ± 4.54 years, and the average years of work experience were 18.94 ± 5.60 years.

#### Expert engagement level

The inquiry was conducted in two rounds, with 18 questionnaires distributed in each round, all of which were returned, yielding a 100% response rate. This reflects strong engagement and a high willingness among the experts to participate. In the first round, 14 experts (77.8%) and in the second round, 6 experts (33.3%) provided feedback, indicating active participation.

#### Expert authority level

In the first round of expert inquiries, the values for Ca, Cs, and Cr were 0.91, 0.83, and 0.87, respectively. In the second round, these values increased to 0.98, 0.92, and 0.95. Both rounds demonstrated Cr values exceeding 0.8, indicating a high level of expert authority and the reliability of the results.

#### Expert opinion concordance level

The coefficient of variation (CV) was used to evaluate the consistency of each individual indicator, with CV values ranging from 0.048 to 0.221 in two rounds of consultation, all below 0.25, suggesting favorable expert consensus on single indicators. Kendall’s concordance coefficient (Kendall’s W) was applied to assess the overall indicator consistency, with coefficients for the importance and operability of items in two rounds being 0.129, 0.137, 0.305, and 0.244 respectively, all statistically significant (*p* < 0.05). These results demonstrate that expert opinions have reached a moderate consensus with gradually improved consistency through multiple rounds of Delphi consultation, and the statistical significance further confirms that such consensus is not formed by random chance.

#### Modified results of solution metrics

After the first round of expert consultations, 14 experts provided 29 revision suggestions. Based on the indicator screening criteria, the expert panel consolidated and modified the suggestions in three key areas: merging “dynamic postural adjustment” and “postural drainage principles” into “postural management”; combining “encouraging patients to cough and expectorate” and “respiratory training” into “airway management”; and consolidating “establishing a research team” and “homogenized training” into “personnel qualification access.” In total, 7 items were deleted, and 21 items were revised or improved. For example, the statement “A decrease in lung ultrasound score indicates effective chest physiotherapy” was revised to “The twelve-zone method was used for lung ultrasound scoring, and a decrease in the score indicated effective chest physiotherapy.”

Following the second round of expert consultations, 6 experts proposed five additional revision suggestions. After discussion and refinement by the research team, the original entries were supplemented and improved. For instance, in the “Position Management” section, the phrase “bed head elevated 30–45 degrees” was modified to “bed head elevated 30–45 degrees in the absence of contraindications.” The final version of the ultrasound-guided CPT protocol for sTBI patients includes four primary entries, 11 secondary entries, and 28 tertiary entries, as detailed in Table 1.

**Table 1:** Ultrasound-guided chest physiotherapy (CPT) protocol for patients with severe traumatic brain injury (sTBI).

First-level item	Sub-item	Tertiary item	Entry importance		Item operability	
			Score ( $\bar{x}$ ±s)	Coefficient of variation	Score ( $\bar{x}$ ±s)	Coefficient of variation
1, Personnel Qualification Access	1.1 Establish a research team	The team members include 3 critical care nurses and 1 critical care physician, all with over 10 years of work experience. They have all undergone standardized training by the Chinese Critical Care Ultrasound Research Group and obtained qualification certificates (34)	4.72 ± 0.46	0.098	4.72 ± 0.46	0.098
	1.2 Homogeneous Training for Team Members	Group members undergo unified training, which includes pulmonary ultrasound operation, ultrasound image interpretation, knowledge and technical training related to chest physiotherapy, as well as learning (16)	4.83 ± 0.38	0.079	4.56 ± 0.51	0.112
2, Pulmonary ultrasound examination	2.1 Fewer than 2 lines on Line A or Line B (indicating good pulmonary ventilation)	2.1.1 Postural management: In the absence of contraindications, elevate the head of the bed by 30°–45°, alternate between left and right lateral positions every 2 h, and maintain the head in the midline position during turning (44).	4.72 ± 0.46	0.098	4.67 ± 0.49	0.104
		2.1.2 Routine airway management to prevent VAP occurrence (15, 19).	4.83 ± 0.38	0.079	4.94 ± 0.24	0.048
		2.1.3 Early progressive passive functional exercises to promote the recovery of pulmonary function and respiratory muscle function in patients (17, 18, 27)	4.50 ± 0.51	0.114	4.39 ± 0.50	0.114
	2.2 Multiple B lines (indicating pulmonary edema or pulmonary interstitial syndrome)	2.2.1 Monitor fluid intake and output, central venous pressure, and right heart function to exclude factors causing pulmonary edema. If pulmonary interstitial syndrome is suspected, promptly administer suction therapy (13, 32)	4.22 ± 0.65	0.153	4.17 ± 0.51	0.123
		2.2.2 Airway management: For patients with artificial airway and mechanical ventilation, regularly monitor the balloon pressure, perform airway humidification, and prevent airway obstruction (19, 23)	4.78 ± 0.43	0.090	4.67 ± 0.49	0.104
		2.2.3 Chest percussion: Focus on enhancing sputum expulsion in the B-line area. Form a bow with your hand and percuss the back (avoiding the cervical region), utilizing wrist strength to strike the patient’s back from bottom to top and from outside to inside. Perform 15–20 min of percussion each time, every 2 h or as needed (30, 41)	4.61 ± 0.61	0.132	4.50 ± 0.86	0.191
		2.2.4 Vibration sputum drainage: The patient is placed in a lateral decubitus position, with a vibration frequency of 20 Hz–30 Hz. Vibration is applied from bottom to top and from outside to inside to facilitate sputum drainage. Each treatment session lasts 10–15 min, twice daily, plus as needed (21, 43)	4.67 ± 0.49	0.104	4.72 ± 0.46	0.098
		2.2.5 Dynamic adjustment: Adjust the frequency and depth of suction based on the patient’s condition, and perform deep suction with fiberoptic bronchoscopy when necessary. Simultaneous monitoring of intracranial pressure is required (33, 37)	4.61 ± 0.50	0.109	4.39 ± 0.50	0.114

	2.3 Fragile sign/tissue-like sign with bronchial hyperinflation (indicating pulmonary consolidation or atelectasis)	2.3.1 Oral care: Perform routine oral care to reduce oral bacteria, thereby decreasing the risk of bacterial ascending infections (25)	4.67 ± 0.49	0.104	4.72 ± 0.46	0.098
		2.3.2 Airway management: Regularly assess the patency of the artificial airway, implement airway humidification and nebulization (temperature 37 °C, humidity 100%), and reduce sputum viscosity (19, 40)	4.78 ± 0.43	0.090	4.50 ± 0.62	0.137
		2.3.3 High-frequency chest wall vibration technique: Wear a vest to cover the entire lung field, 2–3 times/day for 15–20 min, avoiding excessive vibration to prevent increased intracranial pressure (24, 35)	4.39 ± 0.61	0.138	4.06 ± 0.80	0.198
		2.3.4 Postural management: Adjust the drainage method based on the location of atelectasis identified by ultrasound, adhering to the principle of elevating the affected area. Combine techniques such as lung percussion for sputum drainage and nebulization to promote sputum drainage and lung re-expansion (14, 37)	4.22 ± 0.65	0.153	3.94 ± 0.87	0.221
		2.3.5 Gravity-dependent area: When this sign is observed in areas 5 and 6 of the posterior chest wall in both lungs, patients without contraindications for prone positioning should actively adopt prone positioning for ventilation (24, 28, 30) (To be performed when airway safety is maintained, hemodynamic stability is achieved, and intracranial pressure is <20 mmHg.)	4.67 ± 0.49	0.104	4.11 ± 0.68	0.165
		2.3.6 For patients with atelectasis and hyperreversible atelectasis: Perform lung re-expansion therapy. After achieving the target open airway pressure through the PEEP escalation method, adjust the PEEP using the optimal compliance method, and repeat every 8 h (29)	4.33 ± 0.77	0.177	4.11 ± 0.76	0.184
	2.4 Precautions during the inspection	2.4.1 Physical therapy for the chest should be administered 30 min before or 2 h after meals to avoid vomiting during or after the treatment,Less than 30 min (24, 38)	4.78 ± 0.43	0.090	4.61 ± 0.85	0.184
		2.4.2 During thoracic physical therapy, closely monitor the patient’s heart rate, oxygen saturation, respiratory parameters, and intracranial pressure changes, and promptly address any abnormalities to ensure patient safety (39, 42)	4.78 ± 0.55	0.115	4.72 ± 0.57	0.122
2.4.3 If the patient has a head drainage tube, it should be properly secured before performing maneuvers such as turning the patient, back percussion, or vibration sputum drainage. The drainage fluid should be monitored, and any abnormalities should be promptly stopped and addressed (9, 22)		4.78 ± 0.43	0.090	4.78 ± 0.43	0.090
3, quality control	3.1 Quality control of ultrasound images	To ensure assessment consistency, pulmonary ultrasound images were jointly evaluated by two nurses. In case of disagreement, a physician with critical care ultrasound qualifications or above would arbitrate to minimize operator bias (20, 31)	4.83 ± 0.38	0.079	4.33 ± 0.59	0.137
	3.2 Quality control of intervention implementation	3.2.1 The investigator oversees the entire process, with the responsible team leader assuming supervisory responsibilities to ensure that team members conduct evaluations in accordance with established standards and provide assistance when necessary (36)	4.78 ± 0.43	0.090	4.67 ± 0.59	0.127
		3.2.2 The research team performed standardized pulmonary ultrasound evaluation 30 min after the chest physiotherapy intervention to ensure data comparability (26, 30)	4.50 ± 0.62	0.137	4.39 ± 0.50	0.114
		3.2.3 Maintain consistent basic nursing standards throughout the study to ensure research reliability (44)	4.83 ± 0.38	0.079	4.44 ± 0.62	0.139
		3.2.4 The data were uniformly collected by the investigators and then entered into Excel sheets by two individuals for verification to ensure data accuracy and completeness (31)	4.78 ± 0.55	0.115	4.83 ± 0.38	0.079
4, effect evaluation	4.1 Pulmonary ultrasound scoring	The pulmonary ultrasound scoring was performed using the twelve-segment method. If the score decreases without a significant increase in intracranial pressure, it indicates that chest physiotherapy is effective (13, 34)	4.72 ± 0.46	0.098	4.61 ± 0.50	0.109
	4.2 Pulmonary ultrasound findings	4.2.1 A reduction or disappearance of the B line with the reappearance of the A line indicates the effectiveness of chest physiotherapy (12)	4.61 ± 0.70	0.151	4.56 ± 0.62	0.135
		4.2.2 The transition from static bronchial inflation signs to dynamic bronchial signs or a reduction in the area of fragmentary signs/organoid signs suggests the effectiveness of chest physiotherapy (12, 20)	4.61 ± 0.61	0.132	4.61 ± 0.61	0.132
	4.3 Combined with routine imaging examinations such as chest CT	Determine the evaluation effect of pulmonary ultrasound based on routine imaging examinations such as chest CT (35)	4.72 ± 0.46	0.098	4.44 ± 0.62	0.139

### Preliminary application results

#### General information of study subjects

A total of 40 patients with sTBI and 13 nurses were enrolled in the study. Among the patients, 18 were male and 22 were female, with an average age of 61.40 ± 15.06 years. Among the nurses, 11 were female, and 2 were male, with an average age of 28.4 ± 5.7 years.

#### Comparison of outcome indicators between the two groups of patients

Independent-samples t-test revealed statistically significant intergroup differences in Lung Ultrasound Score at all time points within 9 days; significant differences in Clinical Pulmonary Infection Score were only found on day 1; the oxygenation index showed statistically significant differences starting from day 7. After correction for multiple comparisons, no significant intergroup differences in lung ultrasound scores were found on days 1, 3 and 5, whereas the experimental group had significantly lower scores from day 7 onward (*P* < 0.05). No significant intergroup difference was detected in clinical pulmonary infection score. No significant differences in oxygenation index were noted on days 1, 3, 5 and 7, but this index was significantly higher in the experimental group at day 9 (*P* < 0.05). Following independent-samples t-test with multiple comparison correction, no statistically significant intergroup differences were detected in the duration of mechanical ventilation and NICU length of stay. Detailed results are shown in Table 2.

**Table 2:** Comparison of outcome indicators between the two groups of patients.

Group	Time (day)	Control group (*n* = 20)	Experimental group (*n* = 20)	*t*	*P* (unadjusted)	*P* (adjusted)	*Cohen’s d*
Pulmonary ultrasound scoring	1	10.95 ± 2.87	9.35 ± 1.73	2.135	0.041	0.087	0.68
	3	11.65 ± 2.62	9.95 ± 1.96	2.323	0.026	0.071	0.73
	5	11.85 ± 2.91	10.00 ± 1.78	2.428	0.021	0.071	0.77
	7	11.90 ± 2.59	9.60 ± 2.04	3.119	0.004	0.020*	0.99
	9	11.68 ± 2.85	8.52 ± 1.74	4.121	<0.001	0.005*	1.34
Clinical Pulmonary Infection Score	1	5.90 ± 1.83	4.70 ± 1.49	2.272	0.029	0.071	0.72
	3	6.85 ± 1.50	5.80 ± 1.79	2.009	0.052	0.098	0.64
	5	6.70 ± 1.38	6.80 ± 1.51	−0.219	0.828	0.828	−0.07
	7	6.85 ± 1.09	7.25 ± 1.25	−1.078	0.288	0.361	−0.34
	9	7.00 ± 1.30	7.16 ± 1.42	−0.362	0.720	0.816	−0.12
Oxygenation index	1	289.15 ± 115.39	338.55 ± 87.41	−1.526	0.136	0.231	−0.48
	3	317.85 ± 100.15	310.45 ± 86.75	0.250	0.804	0.828	0.08
	5	320.05 ± 95.20	278.95 ± 86.36	1.430	0.161	0.249	0.45
	7	343.45 ± 111.27	265.80 ± 94.67	2.377	0.023	0.071	0.75
	9	365.42 ± 166.23	231.47 ± 59.54	3.307	0.003	0.020*	1.07
Mechanical ventilation duration		8.55 ± 4.11	9.85 ± 3.66	−1.056	0.298	0.361	−0.33
NICU hospitalization duration		10.55 ± 4.53	12.60 ± 5.07	−1.347	0.186	0.263	−0.43

*P* < 0.05, indicating statistically significant differences.

For Pulmonary Ultrasound Scoring and Clinical Pulmonary Infection Score, lower values indicate better outcomes; for oxygenation index, higher values indicate better oxygenation. Cohen’s d = (Control) − Experimental/pooled SD.

#### Nurses’ evaluation of the protocol

In this survey, 13 questionnaires were distributed, all of which were returned, resulting in a 100% recovery and validity rate. The 13 nurses rated the clinical applicability, feasibility, and satisfaction of the protocol at 4.57 ± 0.36 points. All nurses assigned the maximum 5-point score to its clinical significance, unanimously recognizing its considerable value for clinical guidance. Additionally, 89% of the nurses regarded the protocol as applicable and feasible in clinical settings.

## Discussion

### Preliminary scientificity and potential reliability of scheme construction

This research protocol was developed using evidence-based methods, initially constructing a draft of an ultrasound-guided physical therapy protocol for sTBI patients. Expert consultations were subsequently conducted via the Delphi method to refine the protocol, ensuring it was scientifically sound and feasible. The entire process adhered to rigorous scientific research standards. A total of 34 articles were included in the evidence review, including guidelines, expert consensus statements, systematic reviews, RCTs, and quasi-experimental studies, ensuring the scientific robustness of the protocol development methodology. The referenced guidelines and consensus statements were created by authoritative medical societies or professional organizations, further bolstering the clinical applicability and scientific validity of the protocol. The success of the Delphi method relied on the careful selection of experts. A total of 18 experts from across the country participated, representing diverse fields such as critical care medicine, nursing, ultrasound diagnostics, and respiratory therapy, ensuring broad disciplinary and geographical representation. The Cr from both rounds of expert inquiries exceeded 0.8, demonstrating strong overall expert authority. With a 100% questionnaire response rate, this reflects high engagement and enthusiasm among the experts. The CV for all indicators in the second round ranged from 0.048 to 0.221, all ≤ 0.25, and the Kendall’s concordance coefficient was significantly improved in the second round (*p* < 0.001). These findings indicate that expert opinions gradually converged to reach moderate-level agreement across Delphi rounds, which ensures the reliability of the consultation results.

### The constructed protocol exhibits both specialization and goal-oriented characteristics

This study developed a specialized, systematic ultrasound-guided CPT protocol specifically addressing the unique brain-lung interactive injury mechanisms in sTBI patients. Expert consultations were conducted during the formulation process to consolidate their opinions, ultimately reaching a consensus on the importance and feasibility of the proposed measures. The experts showed a high degree of agreement on the core safety thresholds of gravity-dependent area: when this sign is observed in areas 5 and 6 of the posterior chest wall in both lungs, patients without contraindications for prone positioning should actively adopt prone positioning for ventilation, which needs to be implemented on the premise of guaranteed airway safety, stable hemodynamics and intracranial pressure below 20 mmHg, thus establishing a therapeutic safety baseline. Article 2.4.1: Given the high risk of gastric retention and aspiration in sTBI patients, administering CPT 30 min before meals or 2 h after meals minimizes the risk of vomiting during treatment. These protocols were meticulously designed to integrate the pathophysiological characteristics of sTBI patients, ensuring a specialized treatment approach. They strictly adhere to the core principle of “brain protection first,” incorporating clearly defined ICP and hemodynamic thresholds throughout the design. This ensures that pulmonary interventions, such as percussion, vibration, and prone positioning, are implemented within a “safe window” of stable cerebral perfusion pressure and intracranial compliance, thus achieving a dynamic balance between pulmonary rehabilitation and brain protection. The protocol utilizes bedside pulmonary ultrasound as a “visual stethoscope,” enabling real-time assessment of pulmonary conditions through various ultrasound signs (10) (e.g., A-line, B-line, and fragmentation patterns) to differentiate lesion characteristics. This approach allows for precise localization and characterization of pulmonary lesions, facilitating goal-directed nursing interventions. Previous studies (36, 42) predominantly focused on single-link management and lacked systematic approaches for comprehensive care. In contrast, this study introduces four key modules: personnel qualifications, ultrasound evaluation, quality control, and outcome assessment, forming a complete closed-loop management system. Compared to prior studies (28, 30, 34), this protocol not only enables visualized and precise management of pulmonary conditions but also systematically coordinates the balance between pulmonary rehabilitation and brain protection. It provides a scientifically grounded framework for managing sTBI patients, integrating specialized assessment, risk control, and targeted interventions.

### The constructed protocol has preliminary operability and potential clinical applicability

Ultrasound enables real-time, bedside assessment of pulmonary conditions in sTBI patients, providing visual representation of pulmonary lesions based on ultrasonographic findings to guide CPT. This method is user-friendly, highly reproducible, and reduces the human resources, time, and safety risks associated with patient transport. The feasibility of the program was further validated through expert consultations in this study. After two rounds of consultation, the coordination levels of each item in terms of importance (Kendall’s W increased from 0.129 to 0.305) and operability (Kendall’s W increased from 0.137 to 0.244) showed significant improvement (*p* < 0.001). Experts unanimously endorsed the core principles of the protocol, including “brain protection priority,” the integration of ICP and hemodynamic thresholds, ultrasound sign discrimination, and target-oriented interventions. In terms of operability, experts agreed that the implementation, evaluation timing and quality control procedures of the protocol show preliminary applicability and potential scalability in clinical practice. The questionnaire results among NICU nurses showed relatively high scores in clinical applicability, clinical significance, feasibility and satisfaction, which provides a preliminary foundation for the clinical application of this protocol. This protocol transforms traditional empirical evaluations into objective, bedside, real-time, and visualized monitoring, enabling precise localization and qualitative assessment of pulmonary lesions. By dynamically adjusting to ultrasound findings, it establishes a closed-loop management system of “personnel qualification access-intervention implementation-quality control-outcome evaluation,” enhancing the specificity and safety of interventions. Moreover, under the DRG payment model, this technology facilitates precision clinical management, optimizes treatment pathways, effectively controls healthcare costs, and ultimately benefits patients.

### Limitations and prospects

The ultrasound-guided chest physiotherapy protocol for patients with sTBI was developed through evidence-based review and Delphi expert consultation, which has certain rationality and potential clinical practicability. Preliminary application results suggest that this protocol has potential auxiliary value in pulmonary management of sTBI patients. However, several limitations exist in the present study. First, this study employed a quasi-experimental non-randomized design with staggered enrollment periods for the control and intervention groups, which might potentially introduce temporal bias, operator learning effects and shifts in clinical practice, somewhat restricting the strength of causal inference. Furthermore, the evaluation of multiple outcome indicators may elevate the risk of type I error to a certain extent, which could partially affect the reliability of result interpretation. Second, this study was merely a preliminary verification conducted in a single center. Although the protocol was formulated through the Delphi method with expert consultation from experts across the country, validation was performed in a small homogeneous sample of only 40 cases. These factors reduce the external validity and generalizability of our findings, and the applicability of this protocol in other medical centers remains uncertain. Third, no sample-size estimation and statistical power calculation were performed due to the nature of preliminary exploration, which may affect the reliability of the results. At present, we are carrying out large-sample studies in our own hospital, and multi-center research will be conducted in the follow-up stage to further verify the clinical applicability of this protocol.

## Data Availability

The raw data supporting the conclusions of this article will be made available by the authors, without undue reservation.
